# Bereitschaft zur Impfung mit einem COVID‑19-Vakzin – Risikoeinschätzung, Impferfahrungen und Einstellung zu Behandlungsverfahren

**DOI:** 10.1007/s11553-021-00908-y

**Published:** 2021-11-01

**Authors:** Sonja Haug, Rainer Schnell, Anna Scharf, Amelie Altenbuchner, Karsten Weber

**Affiliations:** 1 Institut für Sozialforschung und Technikfolgenabschätzung (IST), Regensburg Center of Health Sciences and Technology (RCHST), Ostbayerische Technische Hochschule (OTH) Regensburg, Regensburg, Deutschland; 2 Research Methodology Group, Universität Duisburg-Essen, Duisburg, Deutschland

**Keywords:** Coronavirus-Impfung, Risikogruppe, Impfnebenwirkungen, Einstellung zu Medizin, Arztbesuche, Coronavirus vaccination, Population at risk, Vaccination side effects, Attitude towards medicine, Physician office visits

## Abstract

**Hintergrund:**

Impfungen stellen eine bedeutende Präventionsmaßnahme dar. Grundlegend für die Eindämmung der Coronapandemie mittels Durchimpfung der Gesellschaft ist eine ausgeprägte Impfbereitschaft.

**Ziel der Arbeit:**

Die Impfbereitschaft mit einem COVID‑19-Vakzin (Impfstoff gegen das Coronavirus) und deren Einflussfaktoren werden anhand einer Zufallsstichprobe der Gesamtbevölkerung in Deutschland untersucht.

**Material und Methoden:**

Die Studie basiert auf einer telefonischen Zufallsstichprobe und berücksichtigt ältere und vorerkrankte Personen ihrem Bevölkerungsanteil entsprechend. Die Ein-Themen-Bevölkerungsbefragung zur Impfbereitschaft (*n* = 2014) wurde im November/Dezember 2020 durchgeführt.

**Ergebnisse:**

Die Impfbereitschaft in der Stichprobe liegt bei rund 67 %. Vorerfahrungen mit Impfungen moderieren die Impfbereitschaft. Sie steigt bei Zugehörigkeit zu einer Risikogruppe. Der Glaube an die Wirksamkeit alternativer Heilmethoden und Befürwortung alternativer Behandlungsverfahren geht mit geringerer Impfbereitschaft einher. Ältere Menschen sind impfbereiter, kovariierend mit ihrer Einschätzung höherer Gefährdung bei Erkrankung. Ebenso ist die Ablehnung einer Impfung mit der Überschätzung von Nebenwirkungen assoziiert.

**Schlussfolgerung:**

Die Impfbereitschaft hängt mit Impferfahrungen und Einstellungen zu Gesundheitsbehandlungsverfahren allgemein zusammen. Die Überschätzung der Häufigkeit ernsthafter Nebenwirkungen bei Impfungen weist auf weit verbreitete Fehlinformationen hin.

## Abstract (en)

**Background:**

Globally, vaccination is an important preventive measure. A pronounced willingness to vaccinate is fundamental for the containment of the Coronavirus pandemic by means of vaccination coverage of society.

**Objectives:**

The willingness to be vaccinated with a COVID‑19 (coronavirus vaccine) vaccine and its influencing factors are investigated using a random sample of the total population in Germany.

**Materials and methods:**

The study is based on a random telephone sample and considers older and previously ill persons according to their proportion in population. The single-topic study on willingness to be vaccinated (*n* = 2014) was conducted in November/December 2020.

**Results:**

The willingness to be vaccinated in the sample is around 67%. Previous experience with vaccinations moderates the willingness to vaccinate. It increases with membership to a risk population. Belief in the efficacy of alternative healing methods and advocacy of alternative treatment procedures are associated with lower willingness to be vaccinated. Older people are more willing to be vaccinated, covarying with their assessment of higher risk in case of illness. Similarly, rejection of vaccination is associated with overestimation of side effects.

**Conclusions:**

Willingness to vaccinate is related to vaccination experiences and attitudes towards health care procedures in general. The overestimation of the frequency of serious side effects with vaccinations indicates widespread misinformation.

Impfungen schützen vulnerable Personengruppen und tragen zur Eradikation von Krankheiten bei. Der Schutzimpfung kommt im Zuge der COVID‑19-Pandemie (Coronavirus-Pandemie) als Mittel der medizinischen Gesundheitsprävention eine entscheidende Bedeutung zu. Der Beitrag untersucht Einstellungen der Bevölkerung und Faktoren, die die Impfbereitschaft mit einem COVID‑19-Vakzin beeinflussen.

## Hintergrund und Fragestellung

Impfungen zählen weltweit zu den bedeutendsten und effektivsten medizinischen Präventionsmaßnahmen sowohl für geimpfte als auch nicht geimpfte Personen [1, 31]. Ärzten/Ärztinnen kommt bei der Verabreichung von Impfungen und damit bei der Durchimpfung eine zentrale Rolle zu. Dabei nimmt die Impfeinstellung von Ärzten/Ärztinnen Einfluss auf die Impfbereitschaft der Patient/-innen [11, 25]. Jedoch werden v. a. bei Erwachsenen notwendige Impfquoten nicht (mehr) erreicht. So lag 2019 beispielsweise für Diphterie und Masern keine ausreichende Durchimpfung in Deutschland vor [20], obgleich die Masern- im Gegensatz zur Diphterieimpfquote seit 2008 stetig ansteigt [19].

Darüber hinaus gibt es weitere Einflussfaktoren, die die Impfbereitschaft krankheitsübergreifend beeinflussen. Beispielsweise hemmen Bedenken hinsichtlich des verwendeten Impfstoffs, eine Geringschätzung des Krankheitsrisikos sowie Misstrauen gegenüber Expert/-innen die Impfbereitschaft mit einer Influenza- oder Masernschutzimpfung. Vertrauen in Expert/-innen und fundierter Kenntnisstand über die jeweilige Impfung und Krankheit erhöhen die Impfbereitschaft [24, 32]. Ebenso weisen Personen generell eine höhere Impfbereitschaft auf, wenn sie Vertrauen in klassische medizinische Verfahren haben [2].

Um die aktuell vorherrschende Coronapandemie und ihre Auswirkungen einzudämmen, wird auch hier eine Durchimpfung der Bevölkerung angestrebt und in der nationalen Impfstrategie vom 06.11.2020 festgeschrieben [4]. Impfstart mit dem ersten zugelassenen COVID‑19-Vakzin Comirnaty war in Deutschland am 27.12.2020.

Befunde zur Impfbereitschaft in der Bevölkerung variieren je nach Studiendesign oder Erhebungszeitraum, wobei für die telefonischen Befragungen stets eine höhere Impfbereitschaft als in den Online-Befragungen berichtet wurde ([28]; Tab. 1).

**Tab. 1:** Übersicht Studien zur Impfbereitschaft mit COVID‑19-Vakzin (Impfstoff gegen das Coronavirus) im Erhebungszeitraum vor der Zulassung und aktuell (Auswahl)

Quelle	Erhebungsmethode	Datum	*n*	Operationalisierung und Ergebnis
COSMO – COVID-19 Snapshot Monitoring [3]	Online	Wöchentlich (seit 03.03.2020), 10./11.12.2020 (Welle 26), 17./18.11.2020 (Welle 27), 29./30.07.2021 & 13./14.07.2021 (Wellen 46 & 47)	Je Welle ca. 1000	*Befragte sollten angeben, ob Sie sich gegen COVID‑19 impfen lassen würden, wenn sie nächste Woche die Möglichkeit dazu hätten.* Impfbereitschaft Wellen 26 & 27: je 54 % „würden sich (eher) impfen lassen“, Wellen 46 & 47: 29 % der ungeimpften Befragten
SOEP-CoV Studie [8]	CATI, Teilstichprobe des sozioökonomischen Panels (SOEP)	04.–07.2020	851 (Tranche Impfmodul)	Nehmen wir an, dass ein Impfstoff gegen das neuartige Coronavirus gefunden wird, der nachweislich keine nennenswerten Nebenwirkungen hat. Würden Sie sich freiwillig damit impfen lassen? Impfbereitschaft 70 % „ja“; nein (abgeleitet) 30 %
ARD DeutschlandTrend November 2020 [9]	CATI	09.–10.11.2020	1504	Weltweit wird derzeit an einem Impfstoff gegen das Coronavirus geforscht. Angenommen, es gäbe einen neu entwickelten Impfstoff gegen das Coronavirus. Wären Sie grundsätzlich bereit, sich gegen Corona impfen zu lassen? November 2020: Impfbereitschaft 71 %: (37 % „auf jeden Fall“ 34 % „wahrscheinlich“) August 2021: Impfbereitschaft 87 % (71 % „bereits mindestens einmal geimpft“, 12 % „auf jeden Fall“, 4 % „wahrscheinlich“)
ARD DeutschlandTrend August 2021 [10]	CATI (dual frame), Online-Befragung	02.–04.08.2021	1312	
Neumann-Böhme et al. (2020) [15]	Online	02.–15.04.2020	7664 (je ca. 1000 aus DNK, D, F, I, NL, P, UK)	Would you be willing to get vaccinated against the novel coronavirus? April 2020 (Deutschland): Impfbereitschaft 70 % „ja“, 20 % „unsicher“, 10 % „nein“. November 2020 (Deutschland): Impfbereitschaft 57 % „ja“, 24 % „unsicher“, 19 % „nein“
Neumann-Böhme/Sabat (2021) [16]		08.–16.11.2020	7115 (gleiche Staaten)	
Vorliegende Studie	CATI (dual frame)	12.11.–10.12.2020	2014	Wenn ein Impfstoff gegen das Coronavirus in Deutschland zugelassen wird: Würden Sie sich impfen lassen? Impfbereitschaft 67,3 % (39,5 % „ja sicher“, 27,8 % „eher ja“) 32,7 % skeptisch/ablehnend (18,8 % „eher nein“, 13,9 % „sicher nein“). Ausgeschlossen: 2,1 % „weiß nicht“, 0,1 % keine Angabe

Im Folgenden wird untersucht, welche Faktoren mit der Impfbereitschaft gegen das Coronavirus SARS-CoV‑2 („severe acute respiratory syndrome coronavirus 2“) vor dem Impfstart in Deutschland zusammenhingen. Dabei werden insbesondere Risikogruppen, die Sorge vor Nebenwirkungen, Erfahrungen mit Impfungen, Einstellungen zu Behandlungsmethoden sowie die Häufigkeit der Inanspruchnahme gesundheitlicher Dienstleistungen betrachtet.

## Studiendesign und Untersuchungsmethoden

### Studiendesign und Stichprobe

Die Studie basiert auf einer von den Autor/-innen in Auftrag gegebenen Ein-Themen-Bevölkerungsbefragung zur Impfbereitschaft. Der bundesweite telefonische Survey auf Basis einer Zufallsstichprobe (Festnetz und Mobilfunk) fand zwischen dem 12.11.2020 und dem 10.12.2020 statt. Der standardisierte Fragebogen enthält 49 Fragen (durchschnittliche Dauer 25 min).

Es liegen 2014 realisierte Interviews vor. Im Vergleich zu Online-Befragungen wird die Altersstruktur in Deutschland besser abgebildet (Altersspanne 18 bis 95 Jahre; der Anteil der Befragten ab 70 Jahren liegt bei 19,9 % in der ungewichteten Stichprobe). Auch Nicht-Internetnutzer/-innen werden befragt [29]. Zum Vergleich: der Anteil der Bevölkerung ab 70 Jahren an der erwachsenen Bevölkerung in Deutschland liegt bei 19,3 % (eigene Berechnung auf Basis der Bevölkerungsfortschreibung zum Stand 31.12.2020 [30]). 51 % der Befragten sind weiblich, 49 % männlich.

### Erfassungsinstrumente

Die Impfbereitschaft wurde mit vierstufiger Antwortskala erhoben (Tab. 1). Für die Auswertung wurde die Variable dichotomisiert (Impfbereitschaft vs. Impfskepsis/Impfablehnung). Die Fragen zur subjektiven Zugehörigkeit zu einer Risikogruppe wurde nach Definition des Robert Koch-Instituts [21] formuliert (alle Formulierungen s. Tab. 2). Fragen zur subjektiven Einschätzung der Wahrscheinlichkeit einer Infektion und zu Konsequenzen einer Erkrankung mit COVID‑19 zielen auf den subjektiv erwarteten Nutzen einer Impfung ab. Da zum Befragungszeitpunkt noch keine Erfahrungen mit einem COVID‑19-Impfstoff vorlagen, wurde nach der subjektiven Wahrnehmung ernsthafter Nebenwirkungen einer Grippeimpfung als Indikator des Wissenstands gefragt. Neben der Zahl der Besuche bei Ärzten/Ärztinnen sowie bei Heilpraktikern/Heilpraktikerinnen im letzten Jahr wurden bisherige Erfahrungen mit Grippe- oder FSME-Impfung erhoben.

**Tab. 2:** Stichprobenbeschreibung und bivariate Zusammenhänge mit Impfbereitschaft

Frage	Antwortkategorien	Häufigkeit (%)	Impfbereitschaft (%)
Ärzte betrachten einige Bevölkerungsgruppen als besonders gefährdet bei einer Erkrankung mit dem Coronavirus. Dazu gehören ältere Personen, Raucher, stark übergewichtige Menschen und Personen mit Vorerkrankungen, wie Herz-Kreislauf-Erkrankungen, chronische Lungenerkrankungen, chronische Nieren- oder Lebererkrankungen, Personen mit einem geschwächten Immunsystem, mit Krebserkrankung oder Zuckerkrankheit. Würden Sie sich selbst zu einer dieser Gruppen zählen? (*n* = 2005; χ^2^-Test *p* < 0,001)	Ja	44,5	77,6
	Nein	55,5	59,2
Wie schätzen Sie die Wahrscheinlichkeit ein, dass Sie sich innerhalb der kommenden 6 Monate mit dem Coronavirus infizieren? (*n* = 1961; H‑Test *p* < 0,001)	Keinesfalls	10,6	45,2
	Wahrscheinlich nicht	39,1	70,4
	Vielleicht	39,1	71,4
	Ziemlich wahrscheinlich	8,7	62,1
	Ganz sicher	2,6	72,5
Wie schätzen Sie die langfristigen Konsequenzen einer Erkrankung mit dem Coronavirus für sich selbst ein? Eine Erkrankung wäre für mich … (*n* = 1990; H‑Test *p* < 0,001)	Völlig harmlos	5,4	19,6
	Weitgehend harmlos	11,3	55,3
	Schwer einzuschätzen	53,5	66,0
	Möglicherweise gefährlich	21,6	84,5
	Extrem gefährlich	8,2	76,3
Haben Sie sich schon einmal gegen Grippe impfen lassen? (*n* = 2006; χ^2^-Test *p* < 0,001)	Ja	59,2	77,5
	Nein	40,8	52,5
Haben Sie sich schon einmal gegen Zeckenbisse impfen lassen? Gemeint ist die Impfung zum Schutz vor Hirnhautentzündungen durch das FSME-Virus (die Abkürzung FSME steht für Frühsommermeningoenzephalitis). (*n* = 1934; χ^2^-Test *p* < 0,001)	Ja	43,2	75,4
	Nein	56,8	62,6
Es gibt viele Belege für die Wirksamkeit von Homöopathie bei der Behandlung von Krankheiten (*n* = 1887; H‑Test: *p* < 0,001)	Stimme überhaupt nicht zu	19,9	80,6
	Stimme eher nicht zu	29,1	70,8
	Stimme eher zu	38,5	64,6
	Stimme voll zu	12,5	47,0
Alternative Heilmethoden helfen bei vielen Gesundheitsproblemen besser als die klassische Schulmedizin (*n* = 1882; H‑Test *p* < 0,001)	Stimme überhaupt nicht zu	18,2	83,4
	Stimme eher nicht zu	38,6	74,0
	Stimme eher zu	29,4	61,7
	Stimme voll zu	13,9	44,5
Die Erfolge von Heilpraktikern werden unterschätzt (*n* = 1845; H‑Test *p* < 0,001)	Stimme überhaupt nicht zu	17,2	75,3
	Stimme eher nicht zu	28,4	74,5
	Stimme eher zu	33,9	66,9
	Stimme voll zu	20,5	49,3
Was halten Sie von … Akupunktur (*n* = 1872; χ^2^-Test *p* < 0,001)	Viel	33,3	61,4
	Etwas	48,4	72,6
	Gar nichts	18,3	68,5
Homöopathie (*n* = 1783; χ^2^-Test *p* < 0,001)	Viel	19,5	46,8
	Etwas	49,1	70,6
	Gar nichts	31,4	76,6
Körpertherapie (*n* = 1801; χ^2^-Test *p* < 0,001	Viel	44,3	62,5
	Etwas	41,3	75,3
	Gar nichts	14,4	65,3
Ayurveda^a^ (*n* = 1136; χ^2^-Test *p* < 0,001)	Viel	14,6	55,8
	Etwas	39,5	70,6
	Gar nichts	45,9	72,8
Bach-Blütentherapie^a^ (*n* = 1197; χ^2^-Test: *p* < 0,001)	Viel	13,3	45,5
	Etwas	28,3	62,7
	Gar nichts	58,4	75,8
Traditionelle chinesische Medizin^a^ (*n* = 1437; χ^2^-Test *p* < 0,001)	Viel	25,4	58,5
	Etwas	44,5	73,8
	Gar nichts	30,1	69,1
Schüßler-Salze (*n*^a^ =1400; χ^2^-Test *p* < 0,001)	Viel	18,2	46,6
	Etwas	38,2	70,0
	Gar nichts	43,6	73,0

^a^Die fehlenden Werte, erkennbar an geringerer Fallzahl, zeigen relativ geringe Bekanntheit an. Aufgrund geringerer Fallzahl wurde der Index nicht in die multivariate Analyse aufgenommen

Zur Erfassung der Einstellung zu alternativen Heilmethoden wurden zwei additive Indizes gebildet. Die Items beider Indizes basieren auf den Studien [7] und [13], die sprachlich dem Fragebogen angepasst wurden. Der Index „Glaube an die Wirksamkeit alternativer Heilmethoden“ besteht aus den Angaben zu 3 Items (Tab. 2). Die interne Konsistenz beträgt Cronbachs α = 0,71.

Der Index „Befürwortung alternativer Behandlungsverfahren“ besteht aus den Angaben zu folgenden Items: Akupunktur, Homöopathie, Körpertherapie (z. B. Kinesiologie, Chiropraktik, Reflexzonenmassage), Ayurveda, Bach-Blütentherapie, traditionelle chinesische Medizin und Schüßler Salze (Tab. 2). Die interne Konsistenz beträgt Cronbachs α = 0,85.

### Auswertungsstrategie und statistische Analysen

Es wird von der Grundannahme ausgegangen, dass die Impfbereitschaft mit subjektiv rational begründbaren Faktoren zusammenhängt. So wird erwartet, dass Personen, die sich zu einer Risikogruppe zählen oder mit hoher Wahrscheinlichkeit ernsthafte Konsequenzen einer Erkrankung erwarten, eine höhere Impfbereitschaft haben und dass bei hohen wahrgenommenen Risiken von Impfungen die Impfbereitschaft sinkt. Fehleinschätzungen in der Bevölkerung würden so die Impfbereitschaft und indirekt die Impfquoten beeinflussen. Darüber hinaus wird untersucht, inwieweit die Impfbereitschaft mit Impferfahrungen, Arztbesuchen und Einstellungen zu medizinischen Behandlungsverfahren zusammenhängt.

Die Auswertung erfolgte mit IBM SPSS Statistics Version 25. Es werden Häufigkeiten und bivariate Zusammenhänge mit der Impfbereitschaft dargestellt (Tab. 2 und 3). Bei den bivariaten Analysen wurde nach konventionellem Skalenniveau ein χ^2^-Test, ein Kruskal-Wallis-Test (H-Test) oder ein t‑Test durchgeführt sowie eine bivariate binär logistische Regression. Die Determinanten der Impfbereitschaft wurden in einem multiplen binär logistischen Regressionsmodell untersucht (Tab. 4). Die Ergebnisse wurden mit einer Irrtumswahrscheinlichkeit von *p* < 0,05 verglichen.

**Tab. 3:** Häufigkeitsverteilung metrischer Variablen und Zusammenhang mit Impfbereitschaft

	Verteilung			Zusammenhang mit Impfbereitschaft	
Frage	Md	M	SD	t‑test	Bivariate binär logistische Regression Odds Ratio (*p*)
Wie alt sind Sie? (*n* = 1995)	51,0	52,0	19,0	*p* < 0,001	1,017 (*p* < 0,001)
Was schätzen Sie, wie häufig treten ernste Nebenwirkungen bei Grippeimpfungen auf? Bitte geben Sie einen Prozentsatz von 0–100 an (*n* = 1842)	26,6	20,0	23,3	*p* < 0,001	0,976 (*p* < 0,001)
Bitte denken Sie an die letzten 12 Monate. Wie häufig haben Sie in dieser Zeit folgende Personen aufgesucht? Allgemeinarzt/-ärztin oder Facharzt/-ärztin (*n* = 1842)	5,4	3,0	9,8	*p* < 0,001	1,023 (*p* < 0,01)
… Jemand, der alternative Heilmethoden anbietet z. B. einen Heilpraktiker/-in (*n* = 1996)	0,4	0,0	1,6	*p* < 0,001	0,87 (*p* < 0,001)

*Md* Median, *M* Mittelwert, *SD* Standardabweichung

**Tab. 4:** Determinanten der Impfbereitschaft (multiple binär logistische Regression)

Unabhängige Variablen	*p*	Odds Ratio
Risikogruppe (Selbsteinschätzung; Referenzgruppe ja)	*p* = 0,094	0,79
Alter in Jahren	*p* = 0,225	1,00
Schätzung d. Wahrsch., sich in den kommenden 6 Monaten mit COVID‑19 zu infizieren	*p* = 0,998	1,00
Langfristige Konsequenzen einer Erkrankung mit COVID‑19	*p* < 0,001	1,65
Eintreten ernster Nebenwirkungen bei Grippeimpfungen (Schätzung Prozentsatz)	*p* < 0,001	0,98
Besuche in den letzten 12 Monaten: Allgemein- oder Facharzt/-ärztin (Anzahl)	*p* = 0,391	0,99
Besuche in den letzten 12 Monaten: Heilpraktiker/-in o. ä. (Anzahl)	*p* = 0,263	0,96
Haben Sie sich schon einmal gegen Grippe impfen lassen? (Referenzgruppe ja)	*p* < 0,001	0,47
Haben Sie sich schon einmal gegen Zeckenbisse impfen lassen? (Referenzgruppe ja)	*p* < 0,001	0,59
Index Glaube an Wirksamkeit alternativer Heilmethoden	*p* < 0,001	0,58

*n* = 1585, χ^2^ 328,9, df = 10, *p* < 0,001, Nagelkerkes R^2^ = 0,279

## Ergebnisse

### Impfbereitschaft

Zum Erhebungszeitpunkt waren 3,2 % selbst infiziert, 12,1 % berichten über Infektionen in der Familie und 17,6 % über Infizierte im Freundeskreis. 72 % haben keine Erfahrungen mit Infektionen. Die Impfbereitschaft mit einem COVID‑19-Vakzin liegt bei 67,3 % (Tab. 1). Bei Impfskepsis oder -ablehnung werden am häufigsten befürchtete Nebenwirkungen als Grund (71,5 %) genannt.

### Risikogruppen

Nach Selbsteinschätzung zählen sich 44,5 % der Befragten zu einer Risikogruppe. Die Impfbereitschaft dieser Personen ist mit 77,6 % um 18 % höher als bei Personen, die sich nicht zur Risikogruppe zählen (59,2 %; Tab. 2). Das Alter hängt mit der Impfbereitschaft zusammen. Das Durchschnittsalter der Personen, die nicht impfbereit sind, liegt mit 47,0 (*s* = 18,2 Jahre) signifikant niedriger als in der Gruppe der Impfbereiten mit 52,3 (*s* = 19,3 Jahre) und dem Bevölkerungsdurchschnitt (Tab. 3). Die Effekte sind unter Kontrolle weiterer Variablen jedoch nicht mehr signifikant (Tab. 4).

### Einschätzung der Infektionswahrscheinlichkeit

Nur wenige Befragte schätzen die Wahrscheinlichkeit einer Infektion im nächsten halben Jahr als ganz sicher oder ziemlich wahrscheinlich ein (zusammen 11,3 %, Tab. 2). Die Mehrheit stuft sich somit nicht als gefährdet ein.

Am höchsten ist die Impfbereitschaft bei Personen, die meinen, dass sie sich ganz sicher infizieren werden. 62,7 % wollen sich ganz sicher impfen lassen, 9,8 % eher ja (zusammengefasste Impfbereitschaft 72,5 %); allerdings gibt es auch unter dieser Gruppe einen Kern von 11,8 % entschlossenen Impfverweigerern. Entsprechend ist die Impfbereitschaft gering bei Personen, die keinesfalls mit einer Infektion rechnen: ein Drittel will sich sicher nicht und weitere 12 % eher nicht impfen lassen. In der multivariaten Analyse ist die Infektionswahrscheinlichkeit kein signifikanter Faktor (Tab. 4).

### Einschätzung persönlicher Konsequenzen einer Erkrankung

Mehr als die Hälfte der Befragten kann die persönlichen Konsequenzen einer eigenen COVID‑19-Erkrankung schwer einschätzen (53,5 %). Ein Drittel hält sie für gefährlich, darunter 8,2 % für extrem gefährlich, 21,6 % für möglicherweise gefährlich. Dies übersteigt den Anteil derjenigen, die die potenziellen Konsequenzen als völlig oder weitgehend harmlos einschätzen (16,7 %). Die höchste Impfbereitschaft ist bei Personen festzustellen, die eine COVID‑19-Erkrankung als möglicherweise gefährlich einschätzen (84,5 %). Wer die langfristigen Konsequenzen als völlig harmlos betrachtet, will sich meist nicht impfen lassen (zusammen 80,4 %). Die erwarteten Konsequenzen beeinflussen die Impfbereitschaft auch im multivariaten Modell (Tab. 4).

### Einschätzung von Nebenwirkungen

Auch bei Impfskepsis oder Impfablehnung würden sich 45,5 % impfen lassen, falls das Risiko von Nebenwirkungen nicht größer als bei einer Grippeschutzimpfung wäre. In der Erhebung wurde das subjektive Wissen über die Auftretenswahrscheinlichkeit von Nebenwirkungen bei Grippeimpfungen abgefragt. 8,5 % können keine Angaben zur erwarteten Nebenwirkungshäufigkeit machen. Der Mittelwert der von den Befragten geschätzten Häufigkeit ernster Nebenwirkungen bei Grippeimpfungen liegt bei 26,6 % (Median 20 %). Erwartungsgemäß sinkt bei einer Überschätzung der Wahrscheinlichkeit von Nebenwirkungen einer Grippeimpfung auch die Impfbereitschaft mit einem COVID‑19-Vakzin, sowohl bei bivariater wie multivariater Analyse (Tab. 3 und 4).

### Besuche bei Arzt/Ärztin oder Heilpraktiker/-in

In den 12 Monaten vor der Befragung wurden durchschnittlich 3 Besuche bei Allgemein- oder Fachärzten/-ärztinnen genannt (Tab. 3). Mit steigender Häufigkeit von Arztbesuchen erhöht sich die Wahrscheinlichkeit zur Gruppe der Impfbereiten zu gehören und umgekehrt unterscheidet sich die Zahl der Arztbesuche von Impfbereiten und Impfskeptischen/Impfablehnenden. Mit steigender Häufigkeit von Besuchen bei Heilpraktikern/-innen sinkt die Impfbereitschaft. Die Zahl der Arztbesuche steigt bei Zugehörigkeit zu einer Risikogruppe signifikant (durchschnittlich auf 6,9; t‑Test 5,806; *p* < 0,001) und Besuche bei Heilpraktikern/-innen sind seltener (durchschnittlich 0,3; t‑Test -2,198; *p* = 0,028). Unter Kontrolle anderer Faktoren verschwindet der Effekt der Arztbesuche (Tab. 4).

### Erfahrung mit Impfungen

Relativ große Bevölkerungsteile haben Erfahrungen mit Impfungen: 59,2 % haben sich nach eigenen Angaben bereits gegen Grippe impfen lassen, 43,2 % gegen FSME. Erfahrungen mit einer Grippeimpfung sind bei Personen mit vielen Arztbesuchen wahrscheinlicher (Odds Ratio 0,919; *p* < 0,001; t‑Test 7,929; *p* < 0,001), wohingegen eine FSME-Impfung nicht mit der Zahl der Arztbesuche zusammenhängt (Odds Ratio 0,989; *p* = 0,742; t‑Test 0,33; *p* = 0,742). Impferfahrungen erhöhen signifikant die Impfbereitschaft mit einem COVID‑19-Vakzin, im Fall der Grippeimpfung von 52,5 % auf 77,5 % und bei FSME-Impfung von 62,6 % auf 75,4 %. Impferfahrungen bleiben auch unter Kontrolle anderer Faktoren Determinanten der Impfbereitschaft (Tab. 4).

### Einstellung zu Behandlungsverfahren

**Abb. 1:**
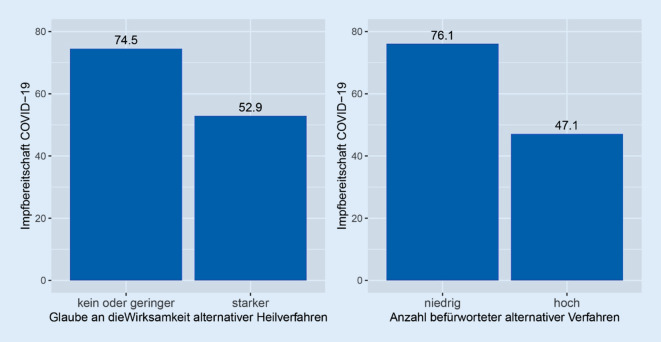
Impfbereitschaft mit COVID‑19-Vakzin (Impfstoff gegen das Coronavirus) nach Glaube an die Wirksamkeit alternativer Heilverfahren (*n* = 1712) und Befürwortung alternativer Behandlungsverfahren (*n* = 718)

Ihre Impfskepsis oder -ablehnung begründen 6,0 % der Befragten mit dem Glauben an Naturheilkunde. 43,3 % aller Befragten stimmen der Aussage (eher) zu, dass alternative Behandlungsverfahren bei Gesundheitsproblemen besser als klassische Schulmedizin helfen würden (Tab. 2). Mit steigendem Glauben an die Wirksamkeit alternativer Heilmethoden sinkt die Wahrscheinlichkeit, zur Gruppe der Impfbereiten zu gehören (Odds Ratio 0,45; *p* < 0,001). Bei Personen mit hohem Glauben an deren Wirksamkeit (oberes Quartil des Index) liegt die Impfbereitschaft bei 52,9 %, bei Personen mit geringem oder mittlerem Vertrauen bei 74,5 % (χ^2^-Test, *p* < 0,001; Abb. 1). Impfskepsis bzw. Impfablehnung ist auch bei multivariater Betrachtung mit dem Glauben an die Wirksamkeit alternativer Methoden verbunden (Tab. 4).

Je weniger die Befragten von alternativen Behandlungsverfahren halten (Index „Befürwortung alternativer Behandlungsverfahren“), desto höher ist die Wahrscheinlichkeit zur Gruppe der impfbereiten Personen zu gehören (Odds Ratio 3,01; *p* < 0,001). Werden viele dieser Verfahren befürwortet (oberes Quartil des Index), liegt die Impfbereitschaft bei 47,1 %, werden wenige alternative Behandlungsverfahren befürwortet, bei 76,1 % (Abb. 1).

## Diskussion

Personen aus Risikogruppen sowie Personen, die die Gefährlichkeit einer COVID‑19-Erkrankung hoch einschätzen, sind häufiger impfbereit. Die subjektive Wahrnehmung von Risiken einer Grippeimpfung, die sich in einer gravierenden Überschätzung der Häufigkeit ernsthafter Impfnebenwirkungen äußert – in der Literatur werden Häufigkeiten von <1/10.000 berichtet [17] –, weist auf ein Informationsdefizit oder Fehlinformationen hin, was wiederum die evidenzbasierte Patientenentscheidung beeinträchtigt [6]. Inzwischen liegen Daten zur Sicherheit der COVID‑19-Impfstoffe vor [22], wobei insbesondere die wechselvolle Diskussion über selten auftretende ernsthafte Nebenwirkungen des Impfstoffs Vaxzevria® von AstraZeneca [12] Bekanntheit erlangte. Die Ergebnisse weisen wie andere Studien auf die Bedeutung zielgruppenadäquater Risikokommunikation hin [14, 18]. Offen ist noch, inwieweit Wissenszuwachs nicht nur die Impfbereitschaft erhöht [5], sondern auch verhaltenswirksam ist. Der Zusammenhang zwischen Impfskepsis und Impfablehnung mit der Befürwortung alternativer Behandlungsverfahren [13] und zwischen Impfbereitschaft und dem Vertrauen in klassische medizinische Verfahren [2] bestätigt Ergebnisse vorheriger Studien zu anderen Erkrankungen.

### Limitationen

Web-Surveys basieren nicht auf Zufallsstichproben und sind daher nicht verallgemeinerbar [27]. Bei methodisch korrekt gezogenen Zufallsstichproben wie der vorliegenden kann im Gegensatz dazu die Ausschöpfung angegeben werden. Die Teilnahmerate in der vorliegenden Studie lag mit 17 % vor dem Hintergrund einer generell sinkenden Teilnahmebereitschaft bei Telefonbefragungen Deutschland in einer üblichen Größenordnung [27]. Auch wenn relativ hohe Ausfälle durch Verweigerung oder Nichterreichbarkeit auftreten, sind diese meist bedingt zufällig (MAR, „missing at random“) und statistisch gut kompensierbar [26]. Zur Anpassung an die Verteilung in der Population wurde ein Gewichtungsfaktor auf Basis des Mikrozensus verwendet.

Die vorliegende Querschnittstudie bildet den Stand vor dem Beginn der Impfkampagne in Deutschland ab. Die Impfbereitschaft ist im Wandel (Tab. 1), ebenso wie das Infektionsgeschehen, die Risikoeinschätzung und Einstellungen im Kontext der Coronapandemie. Unter der Annahme, dass die Zusammenhänge zwischen den Variablen stabil bleiben, ist zu erwarten, dass sich die Impfbereitschaft mit dem Wissen über Risiken einer Erkrankung (z. B. im Hinblick auf Langzeitfolgen auch leichter Erkrankung) oder einer Impfung ändert. Zu berücksichtigen ist auch, dass Impfbereitschaft eine Voraussetzung der Impfentscheidung ist, mit dieser aber nicht gleichgesetzt werden kann.

## Ausblick

Seit der Befragung wurden mehrere Vakzine in Deutschland zugelassen und in Impfzentren, Hausarztpraxen und bei Betriebsärzten/-ärztinnen verabreicht. Der Anteil der vollständig Geimpften wird Ende Juli 2021 mit 57,5 % berichtet [23]. Weiterer Forschungsbedarf ergibt sich zur Entwicklung des Impfgeschehens, insbesondere im Hinblick auf die Impfung von Kindern und Jugendlichen oder Drittimpfungen.

## Fazit für die Praxis

- Die erwarteten Konsequenzen einer Erkrankung oder subjektiv erwartete Impfnebenwirkungen bestimmen die Impfbereitschaft.
- Die Häufigkeit ernsthafter Nebenwirkungen von Impfungen wird erheblich überschätzt.
- Die Risikokommunikation zur COVID‑19-Impfung (Impfstoff gegen das Coronavirus) sollte sich am Wissen der Zielgruppen orientieren.
- Erfahrungen mit Impfungen gegen andere Viren fördern die Bereitschaft zur COVID‑19-Impfung.
- Fehlendes Vertrauen in Impfungen ist insbesondere bei Personen, die i. Allg. alternative Behandlungsverfahren bevorzugen, anzutreffen.
